# “The library is so much more than books”: considerations for the design and implementation of teen digital mental health services in public libraries

**DOI:** 10.3389/fdgth.2023.1183319

**Published:** 2023-07-25

**Authors:** Ashley A. Knapp, Emily Hersch, Clarisa Wijaya, Miguel A. Herrera, Kaylee P. Kruzan, Allison J. Carroll, Sydney Lee, Alex Baker, Alanna Gray, Vann Harris, Robert Simmons, Deepika Kour Sodhi, Nanette Hannah, Madhu Reddy, Niranjan S. Karnik, Justin D. Smith, C. Hendricks Brown, David C. Mohr

**Affiliations:** ^1^ Feinberg School of Medicine, Northwestern University, Chicago, IL, United States; ^2^ Department of Psychology, University of Massachusetts Boston, Boston, MA, United States; ^3^ Department of Psychology, The University of North Texas, Denton, TX, United States; ^4^ Oak Park Public Library, Oak Park, IL, United States; ^5^ Donald Bren School of Information and Computer Sciences, University of California, Irvine, CA, United States; ^6^ College of Medicine, University of Illinois Chicago, Chicago, IL, United States; ^7^ School of Medicine, University of Utah, Salt Lake City, UT, United States

**Keywords:** public library, library workers, adolescents, teens, digital mental health, implementation

## Abstract

**Background:**

Adolescence is a vulnerable developmental period, characterized by high rates of mental health concerns, yet few adolescents receive treatment. Public libraries support adolescents by providing them with access to teen programming, technological resources, and have recently been providing mental health services. Digital mental health (DMH) services may help libraries provide scalable mental health solutions for their adolescent patrons and could be well positioned to address the mental health needs of historically underrepresented racial and ethnic (HURE) adolescents; however, little research has been conducted on the compatibility of DMH services with adolescent patron mental health needs or resource needs of library workers supporting them.

**Methods:**

The research team formed a partnership with a public library, which serves a large HURE adolescent population. We conducted needs assessment and implementation readiness interviews with 17 library workers, including leadership, librarians, and workers with specialized areas of practice. Interview questions focused on library infrastructure, as well as library needs and preferences around the design and implementation of DMH services for adolescents. We used the Consolidated Framework for Implementation Research as guiding implementation determinant framework to code and analyze the interview transcripts.

**Results:**

Our findings revealed library workers play an important role in guiding patrons to desired resources and share a goal of implementing adolescent DMH resources into the library and elevating marginalized adolescents’ voices. Existing library resources, such as the library's role as a safe space for adolescents in the community, close relationships with external and community organizations, and availability of no-cost technological resources, could help facilitate the implementation of DMH services. Barriers related to community buy-in, mental health stigma, and library worker confidence in supporting adolescent mental health could affect service implementation.

**Conclusions:**

Our findings suggest public libraries are highly promising settings to deploy DMH services for adolescents. We identified important determinants that may impact the implementation of DMH services in public library settings. Special considerations are needed to design services to meet the mental health needs of HURE adolescent populations and those adolescents’ most experiencing health inequities.

## Introduction

Adolescence is a developmental period associated with many physical, psychosocial, and environmental stressors as well as opportunities for individuation, growth, and discovery (1). Most mental health disorders originate during childhood or adolescence, and mental health problems experienced during these periods can have ongoing detrimental impacts into adulthood (2, 3). Despite this, few adolescents experiencing mental health difficulties receive care (4, 5). Digital mental health (DMH) services offer the possibility of expanded access to care for adolescents while alleviating barriers, such as time commitments, inconvenience, and stigma (6–9). DMH services consist of a broad range of mental health interventions, programs, and resources delivered through web and mobile-based platforms. They commonly aim to assess, prevent, and treat mental health symptoms, improve well-being, and promote positive health outcomes (10, 11). Many adolescents hold favorable attitudes toward using DMH services and access to technology and broadband is high among this population. For example, in a recent Pew Research Center report, 95% of adolescents aged 13–17 years reported having access to a smartphone and that they are online almost constantly or several times a day (5, 12, 13).

While there is preliminary support for the clinical benefit of DMH services for adolescents, recent reviews suggest insufficient evidence to support the effectiveness of these services (14–16). One reason for this could be that this literature is in its infancy, in that most publications have only reported on acceptability and feasibility. Another reason could be that little attention has been paid to designing implementation plans that consider the broader context, deployment setting, and intended population from the beginning of service design (17). Accordingly, it is possible that current DMH services were not designed to address the unique needs, constraints, and context of deployment settings, and thus are not successful when taken from research studies into the real world (15).

Public libraries offer one promising, yet under-researched, context for DMH service deployment. As an important community gathering place, public libraries offer patrons a variety of services ranging from access to technology and broadband to print and digital collections to community programming (18–22). Libraries also serve as a point of refuge for individuals at risk for or experiencing mental health difficulties by providing a quiet, safe place removed from the everyday stressors that they face in other public or private settings (22, 23). Recognizing their important role in the well-being of their patrons, public libraries within the United States have shifted their priorities to meet patron mental health and social needs (20, 23–25). For example, as a community space that is free and open to the public, libraries often serve adolescents most experiencing inequities and/or from minoritized backgrounds [e.g., underrepresented racial and ethnic (HURE) adolescents] (26). Public libraries have made efforts to create safe spaces for these adolescents by prioritizing diversity, equity, and inclusion (27, 28). In addition to the American Library Association's (ALA) diversity committee who provides materials and programming to public libraries that “*deter hate, foster community, and oppose bigotry toward or oppression against any group*” (26), ALA recently approved a resolution strongly encouraging libraries to provide safe, accessible spaces and services for underrepresented patron populations and to develop relationships with community organizations who can help support those services (21). As another example, most libraries offer mental health trainings for their staff [e.g., Mental Health First Aid (29)], whereas some libraries have even begun to employ social workers to serve the vulnerable populations frequenting the library, who are experiencing food insecurity, homelessness, problematic substance use, and mental health difficulties (20, 23, 25). Other libraries have offered their patrons mental health and well-being related programming (e.g., mental health workshops on topics like suicide prevention and addiction delivered by mental health professionals), as well as both coalited lists and direct access to local mental health resources and services (30).

Few libraries provide DMH services, and those that do, often provide links or membership access to mindfulness apps and other mental health and well-being resources via their websites (23, 31, 32). Further, there is evidence to suggest the majority of adolescents are using public libraries’ digital and non-digital services, and that a significant portion (i.e., 42%) of adolescents and adults who use the library's internet search for health-related information (33, 34). Both statistics emphasize the potential public health impact that library mental health services, and in particular digital services, could have on adolescent mental health. Unfortunately, there is a scarcity of research examining the acceptability, feasibility, and efficacy of mental health services in public libraries, particularly among adolescents and delivered via digital platforms (20, 23).

As public libraries rise to meet their patrons’ mental health needs, some have raised concerns about (1) the difficulty in locating trustworthy and tailored mental health resources and (2) the ambiguity regarding the role public library workers should play, especially those who are not trained social workers (35). Institutional barriers such as lack of employee training, limited funding, and other concerns around how to best integrate mental health initiatives within public library settings have also been raised as significant concerns among library workers. Further, little to no research has been conducted to understand the mental health needs of adolescent patrons and how to best train and support the library workers serving adolescent patrons. Given the high rates of technology, broadband, and library service utilization among adolescents and the library's expansion of both mental health and technological support, adolescent DMH services provided by public libraries are likely to have high uptake and use among this population.

To inform successful implementation in later research stages, it is important to examine contextual determinants in the formative stages of designing a DMH service. In accordance, the research team built a partnership with a public library and conducted needs assessment and implementation readiness interviews with library workers. The goals of the interviews were to better understand the needs and preferences around the design and implementation of DMH services for adolescents embedded within public library services. Specifically, the aims of the current study were to better understand (1) the infrastructure and ecosystem of public libraries, (2) library workers’ needs and preferences in the design and implementation of adolescent DMH services into public libraries, and (3) determinants (i.e., barriers and facilitators) predicted to affect service implementation using the Consolidated Framework of Implementation Research (CFIR) (36).

## Materials and methods

### Setting and participants

This study examines library workers’ perceptions of the implementation of adolescent DMH resources for anxiety within a public library that serves communities in west Chicago, IL and west of Chicago. The library serves community members from diverse racial and economic backgrounds, prioritizing marginalized patrons and those most experiencing health inequities. The library workers consist of leadership (an elected board of trustees, executive director, directors, and managers), librarians, and workers with specialized areas of practice (e.g., Teen Services workers). To protect the identities of those who were interviewed (*N* = 17), we will provide the demographic characteristics of the population who worked at the library at the time of the interviews; notably, interviewees were representative of this population. The demographic data for all workers employed by the public library at the time of the interviews (*N* = 128) were as follows: 62% of library workers identified as female, 35% as male, and 3% as non-binary; and 56% of library workers identified as white, 24% as Black or African American, 12% as Hispanic or Latino, 5% with two or more races, and 3% as Asian. Finally, we will use “*teens*” or “*teen patrons*” to describe the adolescents who frequent the library, as this is the term the library community uses.

### Community partnership

The public library is a social service system for seven communities within and west of Chicago. The library anchors social and public safety services, as well as strategically targets community partnerships and teen development programming. The library's Teen Services program, started in 2019, is specifically designed for teens, by teens, and it focuses on the social-emotional and academic development of teens in the community (see section “Inner Setting” for programming examples). Within the last 5 years, the library expanded its social services to include mental health, responding to the high mental health needs of its patrons. As part of this expansion, the library hired social workers as employees and partnered with an urban medical school to provide free mental health assessments to children, teens, and adults at the library.

It was during this expansion that our team, led by AAK, reached out to the library leadership to learn about the library's recent growth of mental health services and to explore the fit between the library's priorities and research teams’ expertise in DMH services for teen anxiety. A partnership began out of the shared mission of providing accessible, evidence-based mental health anxiety services to teens residing in Chicago and surrounding suburbs, and in particular HURE teens and those who most experience health inequities.

The community-research teams worked closely with the local communities that the library serves through adult and teen advisory boards, informal conversations, formal teen programming, and other partnership activities. We also hired Teen Investigators to help create questions for the advisory board and lead board meetings. Across these activities, teen patrons confirmed interest in receiving mental health support for anxiety via a digital platform at the library. Combining this knowledge with the existing literature suggesting acceptance of DMH services by teens, we then conducted needs assessment and implementation readiness interviews with both library workers and teen patrons focused on the library's ecosystem and considerations of the design and implementation of teen digital mental services for anxiety within the library's programming (12). In the following report we present the results of the library worker interviews; the report on teen patron interviews is forthcoming.

### Procedures

All participants were recruited from the library. Seventeen library workers (identified by participant numbers, e.g., P1, P2, … P17) participated in 1 h-long semi-structured interviews. Recruitment began with library partners from the Teen Services department, who then provided the names and contact information of other workers who had the most contact with teen patrons visiting the library. We also recruited members from the board of trustees, directors, managers, librarians, and workers with specialized areas of practice to participate in the study, to ensure adequate representation of the organizational infrastructure and diverse perspectives. Library workers were provided information about the study and a PDF of the consent form via email prior to participation. All persons recruited expressed interest in participating. Those who agreed to participate provided verbal consent before the interview. Throughout all stages of recruitment and informed consent, the research team underscored the voluntary nature of the study and that participation in the study was not a requirement of their job. All research protocols were approved by the Northwestern University Institutional Review Board before study enrollment.

All interviews were conducted via telephone due to COVID-19 restrictions and library worker preference. Semi-structured interviews asked participants about their roles and workflows in the library, use of technology in the workplace, and library programming focused on teen mental health (see Appendix 1: Interview Guide). Participants were also asked about what resources or features of a digital tool may help them better support teen anxiety at the library and their perceptions of what type of tool may help teens manage their anxiety. All interviews were audio-recorded, transcribed verbatim, and uploaded to Dedoose (37). The interview guide was developed via an iterative process between our team with expertise in implementation science, social work, digital mental health, human-computer interaction, community-engaged research, library science, and adolescent development. The ultimate goals while developing the interview questions were to better understand the workflow and routines of library workers as well as their current and desired teen mental health resources.

### Data analysis

Two researchers conducted a qualitative analysis of the transcribed library workers’ interviews. These coders both had master's degrees, with one also having a doctoral degree, and training in qualitative analysis. Data analysis was guided by CFIR, an implementation science determinant framework that organizes constructs that positively or negatively impact service implementation in five domains: Intervention Characteristics, Outer Setting, Inner Setting, Individual Characteristics, and Process [for more details see (36)]. CFIR provides a comprehensive taxonomy compiled from various related disciplines (e.g., psychology, behavioral economics, organizational change, sociology) that is well-suited to capture the complexities of contextual determinants that are likely to influence the implementation effectiveness of adolescent DMH services into libraries (36). Due to its flexibility, the CFIR is also well-matched to capture the barriers and facilitators of the pre-implementation of an intervention in novel community settings, such as public libraries, as it is common to adapt CFIR for specific, complex settings (38–41). At the outset, the codebook consisted of all domains from CFIR. Using an iterative approach of codebook refinement, coders first read through five interview transcripts each and applied relevant codes from CFIR independently. Then coders met to discuss code application, resolve discrepancies in how codes were being applied, and refine the codebook accordingly (i.e., some code definitions were refined and made more precise; some codes were collapsed due to high overlap). Next, coders applied codes to two additional interview transcripts with the updated codebook, after which they met to resolve discrepancies and further refine the codebook by removing codes that were either underutilized or not present within the transcripts (i.e., codes from Intervention domain). After these two iterations, the final codebook, comprising codes related to the Inner Setting, Process, and Outer Setting domains, was used for code application on all transcripts. Throughout the coding period coders met weekly to ensure coder consistency. Once coding was completed, the codes were grouped by CFIR domains in a hierarchical fashion and were analyzed to decipher implementation determinants. The valence of determinants (i.e., barriers/facilitators) was inferred from how participants referenced each determinant in interviews. In the manuscript review process, it was discovered that two determinants originally classified within the Structural Characteristics construct (Inner Setting domain) were better suited for the Knowledge and Beliefs about the Intervention and Self-Efficacy constructs (Characteristics of Individuals domain).

## Results

Below we organized the results into the four CFIR domains that were identified through coding: Inner setting, Process, Characteristics of Individuals, and Outer Setting (see Table 1 for determinants by CFIR domain) (36). Coding revealed no references to the Intervention domain. We **Capitalized and Bolded** the main constructs (e.g., **Culture**) within each CFIR domain and ***bolded and italicized*** the subconstructs (e.g., ***compatibility***). To provide context for participants’ comments, we included their primary role within the library setting and their participant number along with direct quotes. Library worker roles fell into three main groups: (1) librarians, (2) specialized areas of practice (e.g., Teen Services workers; social workers; front-facing workers), and (3) leadership (e.g., managers; directors; executive director; elected board of trustees).

**Table 1:** Implementation determinants within the consolidated framework for implementation research's domains and constructs.

CFIR domains and constructs	Implementation determinants
I. Outer setting domain	
A. Cosmopolitanism	**Barriers** —
	**Facilitators** - Strong local network due to tight connections with local external organizations and entities, such as governmental agencies (e.g., schools, park districts, etc.) - Connections to free community mental health clinics to refer for higher risk mental health concerns - Intergovernmental agreements (e.g., interventionists working with teens at the library)
B. Community needs and resources	**Barriers** - Lack of teen-specific mental health resources - Stigma and confidentiality (especially with caregivers) around teens’ seeking mental health resources - Skepticism from the community on the role libraries play in mental health and potential overreach
	**Facilitators** - Evolution of library services to include mental health in response to community needs - Library workers’ openness to discussing community needs such as economic disparity, homelessness, and other concerns related to mental health
II. Inner setting domain	
A. Structural characteristics	**Barriers** —
	**Facilitators** - Librarians and library workers in specialized areas of practice serve as a conduit in connecting patrons to mental health resources - Employment of social worker(s) that can attend to higher risk mental health needs
B. Implementation climate	**Barriers** —
	**Facilitators** - High Compatibility between public library and research team on provision of accessible and evidence-based mental health services to teens - Prioritization of teen voices and their needs around mental health - High leadership support for, and interest in, implementing teen digital mental health services into the library - Availability of broadband, computers, and other technology-based resources for free to library patrons
C. Culture	- **Barriers**- Some previous programs not designed to meet the needs of HURE teens, and often further marginalize these teens
	**Facilitators** - Library workers’ deep investment, devotion, and reach they have in the community - Libraries’ core values of honoring, empowering, respecting the voices of all library patrons - Library workers’ empathizing with teens as they navigate the transitional stage of adolescence, especially teens experiencing vulnerabilities such as food insecurity, racism and racial injustice, neighborhood disinvestment, and other hardships - Investment in the library's cultural values of restorative justice, equity, and antiracism (e.g., restorative justice practices like Peace Circles) - Intentionally creating safe spaces for marginalized communities (“Queerios”, named by teens, created for LGBTQIA + teens and allies)
D. Readiness for implementation	**Barriers** - Low employee availability to attend trainings and low knowledge of trainings offered
	**Facilitators** - “*Learn While You Earn*” program that compensates library workers for completing trainings (e.g., mental health first aid) - Strong leadership interest and commitment to service implementation - High alignment of the library's mission and strategic plan with implementation of digital mental health services for teens
III. Characteristics of individuals domain	
A. Access to knowledge and information	**Barriers** - Library workers’ not knowing where and how to identify effective and teen-specific mental health resources - No formal process or centralized protocol in responding to low- and high-risk mental health concerns
	**Facilitators** —
B. Self-efficacy	**Barriers** - Library workers’ low confidence and limited training in mental health, especially teen mental health
	**Facilitators** —
IV. Implementation process domain	
A. Engaging	**Barriers** —
	**Facilitators** - Current staff infrastructure to fill the roles of formally appointed implementation leaders, champions, and other key stakeholders

### Inner setting

**Structural Characteristics** refer to descriptions of the social architecture, age, maturity, and size of the organization where the service will be provided. A central feature and facilitator of the library's social architecture is the overarching function the library and its workers play as a conduit in guiding patrons to desired resources. This structure has important implications for how library workers interact with teens, their imagined role in DMH services, and how teens may expect to engage with services. For example, P14 (leadership) describes, “*I think that that's something we all, who work with the library, have to realize, that we're not going to be the problem solvers. We're the people who push people or show people where they can go to find solutions*.”

For successful implementation, it is important to understand the library's receptivity of service implementation, or **Implementation Climate**, and to ensure that community and research teams’ priorities and expertise align. Library workers and the research team realized in their first meeting that they were united in their mission to provide accessible and evidence-based mental health services, particularly those related to anxiety, to their community of teens. This suggested high ***compatibility***, or a great fit of meaning and values related to the provision of teen mental health services between the community and research teams, which was echoed in the interviews. P11 (specialized area of practice) spoke to compatibility, “*I think its [the library's] commitment to doing something about the stress and anxiety for the mental health of youth and teens has evolved rather significantly over the last few years*”, and P9 (specialized area of practice) spoke to the shared vision, “*I love the idea of having a place—like if a teen comes in having anxiety, to be able to say*, ‘*Hey, look, we have this available. It's specific for teens*’.”

The **Culture** of the library, and the values library workers shared, involved deep investment in and reach within the surrounding community, serving as strong implementation facilitators. P13 (leadership) underscores the library's core values by saying:

“*the vision being empowering every voice in the community, making sure that people are heard, people are honored, people are respected, people are afforded the dignity that they deserve, and giving people an opportunity through what the library does to share their voice*.”

Library workers’ empathy for teens and the difficult developmental stage they were navigating was palpable when they talked about their job and the interactions they had with teens. As P12 (librarian) mentioned,

“*I appreciate being considered as somebody who works with the public, but in the end for me, at least, I always want it to be mostly about the teens… I just want to be able to be of service and make things easier for them. Because being a teen is hard and I feel like we don't focus on that enough…*”

Library workers talked about empathizing with teens as they navigate this transitional stage, such as issues related to gender identity and race, and figuring out who they were/wanted to be. Library workers also underscored the hardships specific to the population frequenting their library, as not only are some of the teens going through the transitional stage of adolescence, but as a vulnerable population they are often experiencing food insecurity, racism and racial injustice, economic disparity, homelessness, neighborhood disinvestment, and other hardships.

Restorative justice, equity, and antiracism were mentioned frequently as core cultural values of the library and were integrated into their services. One example of restorative justice practice was the Teen Service's activity of Peace Circles, which invites all to share and comment respectfully, focused on promoting equity. P4 (librarian) provides another example,

“*I think that they have several people in place [in the library] that are used to working with teens and are coming from a restorative justice background … in that the first option is not to call the police or to call security but instead to talk to them, to convince or persuade them to not be disrespectful or disruptive, and to really try to get at the core as to why they're doing what they're doing*.”

Restorative justice was practiced at many levels, ranging from informal and formal restorative practices, to not escalating situations with police or security intervention.

As for equity and antiracism, for some library workers this was a new journey of learning about the whiteness of the library institution at the national and local levels. Understanding this phenomenon resulted in the realization that some programs were not designed to meet the needs of HURE populations of teens and, inadvertently or not, often further marginalized those teens. Other library workers described hearing directly from teens. For example, P13 (leadership) said:

“*what I hear is that, from Black and Brown people specifically, but also from Black and Brown youth more specifically, that we need to listen more to what their needs are, the ways that they want to use the library … the opportunities that they need for interaction, and that then becomes the need to understand and perhaps change how we're doing things*.”

Experiences such as these have led library workers to reflect on whose voices are being heard and prioritized, understanding that the answers to these questions determine the programs and services that are prioritized and funded. In reference to our community-research partnership, a library worker (P16; leadership) encouraged our team, “*as you're doing the work, you need as much as possible try to center Black voices … Too many times, work in [Chicago community] has left those voices to the side, particularly for the youth*.”

A final core value held by the library organization and its workers is ensuring the library is a safe space for all, especially those from marginalized communities. P9 (specialized area of practice) underscores this value, “*I am huge on giving people a place to go that it's a haven. It's one of the things I love about libraries is it definitely does that*.” Examples of safe spaces mentioned by library workers were a program in collaboration with local schools called Queerios which was named by teens and created for LGBTQIA + teens and allies; the library's partnership with local barber shops that provide free or discounted haircuts; and a program led by a local social justice activist called Living History, focused on helping “*young people with social-emotional learning as well as to create critical consciousness of different issues* [P7; librarian].” Living History has been described as “*a safe space, particularly for the people of color in a community that doesn't have a lot of safe spaces for people of color*” (P12; librarian). In sum, library workers strive to make the library a place where teens from all walks of life feel like they belong, have designated safe spaces, and personal connections with trusted adults.

**Readiness for Implementation** and ***leadership engagement***, or the commitment and involvement of the library and library's leadership to service implementation, is critical to successful and sustained implementation. Across interviews, and particularly those with leadership roles, there was resounding support for, and interest in, embedding teen DMH services for anxiety into existing library programming. This suggests that robust leadership interest and commitment can function as strong facilitators. Upper management indicated that they and other leaders at the library “*consider this work to be very important*” (P15; leadership) and expressed their excitement about the community-academic partnership, saying “*the timing of the opportunity for us to partner with you all is fantastic*” (P2; leadership). Another library worker in a leadership role said, “… *from my perspective, I think it's amazing and a great thing that we can work together on important things like this—to get some of these things accomplished*” (P13; leadership). When asked if the community-academic partnership and mental health tools we propose to co-create fit within the library's mission and strategic plan, a library worker in a leadership role responded, “*I think it fits in very well. What we've been … looking at [is] how public libraries are evolving to provide services and resources to the communities in different ways that meet the communities where they are*.” This person went on to say, “*I can definitely see those kinds of tools [teen DMH services] being very useful*” (P15; leadership). Finally, a person in a leadership position emphasized, “*I've seen it could really turn into a whole kinda mental health type of program, right, where it's like that could be an area of focus…*” (P2; leadership). They go on to say it will simply be a task of integrating it into the existing Teen Services programming and scaling it out to its own type of program.

Several ***available resources*** at the library could facilitate the implementation of DMH services. First, the physical library itself is a central hub for teens in the community, and thus has a broad reach within the teen community. P16 (leadership) stated, “*But [there is] no place, you know, for kids to go after school and hang out and live and just be. And what that has created is that the library has become that*.” Given the large influx of teens at the library, another facilitator is the infrastructure investment to support teen activities and programming. Examples include the library's recent expansion of a physical space designed by and specific for teens, as well as the recent growth of the Teen Services team to include additional roles (e.g., Teen Services Coordinator; see Process domain).

There are several technological resources available to both library workers and teens at no charge within the library that could serve as facilitators in accessing DMH tools. In addition to free broadband, teens can reserve devices like laptops, iPads, and Chromebooks, as well as accessories like a phone or computer chargers and headphones. Library workers mentioned iPads as the most popular item for teens to use at the library and that iPads were frequently used in teen programming activities.

Regarding available resources for library workers, the library has a “*Learn While you Earn*” program that could serve as a strong facilitator in implementing services around DMH tools. This program compensates library workers who complete professional development opportunities, including webinars and training (e.g., mental health first aid; trauma-informed care) that are run by library workers. While these are excellent resources, especially those for mental health, some library workers were not aware of these mental-health specific trainings or noted they “*don't have the time or availability to take those workshops and trainings*” (P5; specialized area of practice).

Finally, funding and where it originates is important to consider when thinking of implementing services into public libraries, especially those services that will require additional funds to launch and/or maintain services after research funding ceases. P13 (leadership) describes, “*The overwhelming majority of the funds that the library has to operate come from local real estate taxes*.” Leadership also mentioned that public libraries have access to local and federal grants, many via the American Library Association, to supplement, maintain, and even launch new programs. If a library is experiencing limited funding, or low allocation of funding toward teen mental health services, grants may be an effective strategy to overcome this barrier. Alternatively, grants could serve as a facilitator in libraries where overall funding, or funding allocated to teen mental health services, is limited.

### Characteristics of individuals

To achieve implementation goals, capturing the characteristics of library workers, including their **Knowledge and Beliefs about the Intervention** and **Self-Efficacy** in executing implementation goals, is critical. Given the library's social architecture and the roles of library workers to serve as a conduit or facilitator of services, two major barriers to implementing future DMH services were identified in our interviews: (1) lack of confidence and training in teen mental health, and (2) lack of knowledge about where and how to identify effective and teen-specific mental health resources. P10 (specialized area of practice) stated,

“*but in the library world in general, is that we're aware of the fact that we are not trained to deal with people who are struggling with mental health issues…I would very much like to learn more—as much as possible before being placed in a position where a teen comes in, and they're dealing with some type of abuse, and I don't know how or what to say or what to do about it*.”

The need for an explicit process for addressing teen mental health concerns, and related mental health training, was echoed by other library workers. They described their need to consistently and collectively know what to say and do when mental health concerns are shared by teen patrons, especially high-risk mental health concerns (e.g., suicidal ideation; abuse). They also expressed a desire for a centralized protocol outlining the recommended actions different roles should take (e.g., role of librarians vs. social workers).

### Process

To understand the potential for DMH service implementation within the library, it is important to identify library workers that are well positioned to implement, market, and support the service, referred to as **Engaging** within the CFIR framework. To do so, library workers reflected on their roles within the organization. Teen Services workers (i.e., Teen Services Manager, Coordinator, Specialist, and Librarians) had the most direct interaction and deepest relationships with teens developed through informal conversations and planned programming; accordingly, library workers felt they would be best positioned to lead the implementation of the service—i.e., as the ***formally appointed implementation leaders***. As such, the Teen Services workers would have the primary responsibility of implementing the DMH resources. P6 (specialized area of practice) underscores this unique role of Teen Services workers saying, “*Staff who are experts in working with teens, and that's their job. That's their role. They have daily contact… [and] have longer relationship-building type of contact*.” As for ***champions***, or the main supporters and marketers of service implementation, interviews suggested persons who serve as security guards, front-facing workers, middle school services workers, and the marketing/communications teams could be best for this role. They can support and market service implementation because they have a wide reach via regular personal interactions with caregivers and teens (e.g., providing book recommendations) or through communication outlets like the library's weekly e-newsletter. Directors, managers, and elected trustee board members have more limited interactions with teens, primarily learning about teen mental health concerns via their team or if a concern (e.g., suicidal ideation) is escalated to them. The board's primary roles are to set policy, approve the budget, and appoint the executive director. It was discussed that those in these leadership roles would be considered ***key stakeholders***, as they have strong influence on what is implemented and financially supported in the library.

### Outer setting

One of the most prominent facilitators in the Outer Setting described by library leadership and workers is the library's tight connection to external organizations, referred to as **Cosmopolitanism**. These facilitators speak to the strong network the library has created with local entities and the wide reach of the library. For example, the library contracts prominent members, such as activists and artists, in the surrounding communities to either lead or assist with teen programming (e.g., LGBTQIA + group). Additionally, the library has a tight network with other governmental agencies, such as local schools, park districts, and free community mental health clinics. Library workers do outreach each week at local schools to promote upcoming activities for teens (and their caregivers) at the library. Other connections with external organizations are created through intergovernmental agreements, where the library pays into a service that is then part of the library programs. For example, interventionists working with teens who are licensed social workers from a local teen services organization come to the library for several of the teen services throughout the week and are also available for mental health crisis referrals. P13 (leadership) provided a description of the library's connections with external organizations as, “*So, whether it's a formal or informal kind of agreement, there's a lot of expectation in the community that these organizations are going to work together, and so there's a lot of commitment from the organizations that do that*.”

The extent to which the **Community Needs and Resources** are realized and prioritized by the library will be important when transitioning to implementation from service design and effectiveness testing. Library workers indicated the services they offer are in response to their community's needs; traditionally, these needs have been related to literacy and book/media access, but within the past decade, library workers discussed the evolution and expansion of community needs. As P2 (leadership) indicated, “… *there's just a significant [portion] of our patrons [persons who use the library] that they need a little more than just the resources around just collections*.” This library worker went on to say that a significant number of community members come to library workers to discuss their experiences with economic disparity, food insecurity, homelessness, and problems related to mental health. In fact, needs around teen mental health were described as one of the largest needs library workers heard from teens. While stress and anxiety were the most common mental health struggles teens expressed to library workers, there was vast diversity in the circumstances affecting their mental health.

For example, teens’ stress and anxiety ranged from their experiences of racism and racial injustices, to pressure for high academic achievement, to navigating relationships with their family, friends, and romantic partners. The heterogeneity of teen experiences related to stress and anxiety underscores the critical need for diverse teen voices to be at the design table to ensure the creation of digital tools and services meet the specific mental health needs of teens who frequent the library. P5 (specialized area of practice) underscored this important takeaway,

“*I think that teens of different economic level and different backgrounds respond differently to stress and anxiety and mental health or are facing different challenges, too… it's important to account that each one is facing their own challenges based on their own specific circumstance, and it may be kinda hard just to paint everything with a broad brush*.”

Additionally, library workers reported that teens have voiced their frustration about finding resources to help them with their mental health wellbeing. In addition to access, P2 (leadership) highlights other critical barriers to accessing teen mental health resources, “*the stigma and also access—they [the teens] just felt that there weren't many access or tools that they could reach out to, really without their parents knowing, too*.”

While the library has prioritized and taken action to meet the community's mental health needs (e.g., hiring social workers, working with local universities to offer free mental health assessments), library workers also described the library's place or role in meeting the community's mental health needs as a point of debate within the library and broader community. As described by P14 (leadership), “*some people have been critical of the library in recent years of overreach… Is this really what the library should be doing? Should the library be doing mental health stuff*?” This quote highlights some of the community's skepticism of the library prioritizing mental health concerns. Along with the implementation barrier of community buy-in, community concerns around overreach also speaks to the broader tension within libraries of managing competing community needs.

## Discussion

Given the research-to-practice gap in which numerous efficacious DMH services have failed to be sustainably implemented in real-world settings, partnering with the deployment setting and its service providers in both the design of services and blueprints for implementation is critical (42, 43). This partnership is even more critical when designing and implementing services with marginalized and HURE populations, as these communities have historically been un- or under-represented in the design, resulting in services that are not designed to meet their unique needs (e.g., coping with distress due to racism; marginalization) (44, 45). Results from our needs assessment and implementation readiness interviews with library workers gleaned critical information on the library ecosystem, specific determinants (facilitators and barriers) to implementing teen DMH services into public libraries that serve marginalized and HURE populations, as well as library worker needs for successful design and implementation of these services.

*Facilitators* specific to function, compatibility, and reach suggest public libraries are promising settings for the effective implementation of DMH services for HURE teens and those most experiencing health inequities. Interview findings suggested that libraries and its workers serve as conduits, in that one of its main functions is to guide community members to their desired resources and services. Further, library workers spoke about the recent expansion of their services to include mental health and underscored the high compatibility and shared priorities between the public library and the research team. In particular, the shared priorities were to provide accessible, evidence-based mental health services for anxiety and stress, as these were the most common mental health concerns raised by teen patrons to library workers. As evidenced by our results and the extant literature, public libraries are promising implementation contexts because they serve, and have earned the trust of, marginalized and HURE teen populations that have not found safe spaces elsewhere in their communities (26). This highlights public libraries’ unique, broad reach to important communities who have been underrepresented in mental health service design and provision. As a next step in our community-research partnership, the research team will work closely with library workers and teen patrons to design a DMH service, test service effectiveness, and evaluate implementation process and outcomes. Taking advice from library workers, we will prioritize centering the voices of Black and other historically underrepresented populations of teens throughout these processes as well as build off of the innovative DMH work being conducted with HURE populations (46–50).

Feedback from library workers also underscored important *barriers* to consider for effective implementation, and in particular, for reach and adoption of teen DMH services into libraries. First, as part of the Outer Setting, community buy-in was identified as an important barrier, in that some community members expressed concerns about library overreach regarding the provision of mental health services. Given that community priorities inform where funding is allocated within the library, shortage or lack of funding allotted to mental health resources could arise as a barrier if such community concerns are not addressed. Second, library workers heard from teens that mental health is stigmatized, deterring teens from inquiring about relevant resources, and that most resources were not specific to teens and often gate-kept by caregivers and other adults. The latter encourages continued discussion on ethical considerations of direct provision of mental health resources to teens, from both perspectives of caregiver consent and withholding resources from vulnerable teens in crisis.

As for *barriers* specific to library workers serving as conduits, several workers expressed not feeling confident and/or having a lack of training to effectively address mental health concerns brought up by teens, which is consistent with other reports on library worker experiences in response to patron mental health needs (25, 35). Library workers also mentioned not knowing where to locate effective and accessible teen mental health resources, especially those designed or tailored for marginalized and HURE teens. Fully automated DMH services may fit well with the main purpose of libraries connecting patrons to resources, in that patrons could be referred to commercial or academic digital interventions that fit their needs to “*check out*” for free, similar to how books and e-books have traditionally been accessed in libraries (31, 32). This route may be most successful for lower-risk mental health concerns, compared to high-risk concerns (e.g., suicidal ideation; food insecurity), of which may need to be triaged out to social workers at the library or local community mental health clinics in their tight network. As libraries expand their role to include the provision of mental health services and resources, coaching or providing “*human support*” for digital services could also be considered (51). In addition to increasing the likelihood of successful uptake and engagement by teens, training to be a coach could also help in equipping and building the confidence of library workers in addressing teen patron mental health concerns. If coached services are offered by public libraries, addressing concerns related to library worker training, knowledge, and burden related to service provision will be critical.

### Limitations

These findings and implications should be considered within the limitations of this study. First, the interviews and data analysis were conducted by persons who identify as white. It is possible our views as white persons may have led us to deemphasize, overlook, or misinterpret aspects of the data, especially as it relates to topics such as equity, antiracism, and racial justice. The researchers took measures to reduce bias by engaging in reflexivity, reaching researcher consensus throughout data analysis, member-checking with library workers, and including library workers as co-authors who reviewed and edited this report (52). Second, the public library we worked with was well-resourced with existing social services and several library workers passionate about teen mental health. In particular, they have employed social workers, allocated budgetary resources to mental health and teen services, and have close collaborations with other local organizations that provide teen mental health services as well as with medical universities to provide free mental health assessments to patrons. Thus, this public library may not be representative of the needs of other libraries with different infrastructures and resources; however, the steps this public library took could be considered by other libraries as preliminary work to support future implementation of DMH resources. Third, coding, analysis, and write-up began prior to the release of the updated CFIR 2.0 (53). Given this and that the main changes to the constructs identified from CFIR 1.0 to CFIR 2.0 were recategorization, we elected to maintain CFIR 1.0 as the guiding framework. Further, only four of the five CFIR 1.0 domains were represented in the responses. One reason could be that we were early in the intervention design process and had not yet solidified the DMH service to be implemented at the time of the interviews, such that the Innovation Characteristics domain would not be as relevant. Another reason could be that our interview guide was not designed from the CFIR interview guide tool, rather it was a formal assessment of library infrastructure and worker roles, as well as library worker preferences on the design and implementation of a teen DMH service for anxiety within their library and then coded for CFIR domains and constructs. The Innovation Characteristics CFIR domain is critically important and a content area that we are in the process of collecting through other data. Finally, the needs assessment and implementation readiness interviews were conducted during the first year of the global pandemic. While we do not believe this to limit our data, it is a historical context that should be considered when reading and interpreting the findings, especially since the needs of mental health for youth have increased (54–56).

### Conclusion

Most often digital services are created and tested with little involvement from the community, especially with teen and HURE communities, as well as with little consideration of deployment settings. These are likely large contributors to the gap we see in efficacious digital services failing in real-world environments (57). If our research community is to create services that are both effective and sustainable, we must design services with the intended community for specific settings. Our results suggested public libraries are highly promising settings for the deployment and sustainment of teen DMH services focused on marginalized and HURE teen populations. We also received critical feedback from library workers on specific design and implementation considerations of these digital services. Our community-research team's next steps will involve working closely with teens, and in particular marginalized and HURE teens, and library workers to co-design services and to evaluate the implementation process and outcomes (42, 58, 59).

## Data Availability

The raw data supporting the conclusions of this article will be made available by the authors, without undue reservation.

## Appendix 1: Interview Questions

***Around their Role***

• How long have you worked at the library?
• I'm interested in understanding your daily workflow/routine. Could you tell me more about your daily routine?
• What kind of technologies do you use in your workplace? Are any of those tools mandated for you to use in your day-to-day work?
• Broadly, how do you feel about using technology-based resources or tools in your work?

***Mental Health Specific***

• What are your perceptions of library's support of anxiety and stress of youth?
• What types of problems do teens at the library express to you related to their stress and anxiety?
• Do you have time in your work schedule reserved to talk to youth about their mental health, such as stress and anxiety?
• How do you respond to their concerns related to stress and anxiety? Is it something you feel you can respond to or do you direct youth to someone else?
• Does the library provide training on mentoring youth with mental health concerns, like anxiety and stress?
• What resources would be helpful to you as you listen and provide guidance to teens at the library on stress and anxiety?
• How could a digital tool that helps youth manage their anxiety be best incorporated into the services library offers its youth patrons?
• How could you personally incorporate this digital tool into your interactions with youth during your daily work routine?
